# The MAPK Signaling Pathway Presents Novel Molecular Targets for Therapeutic Intervention after Traumatic Spinal Cord Injury: A Comparative Cross-Species Transcriptional Analysis

**DOI:** 10.3390/ijms222312934

**Published:** 2021-11-29

**Authors:** Mohammad-Masoud Zavvarian, Cindy Zhou, Sabah Kahnemuyipour, James Hong, Michael G. Fehlings

**Affiliations:** 1 Division of Genetics and Development, Krembil Brain Institute, University Health Network, Toronto, ON M5T 2S8, Canada; Mohammad.zavvarian@mail.utoronto.ca (M.-M.Z.); cindym.zhou@mail.utoronto.ca (C.Z.); jmsyl.hong@gmail.com (J.H.); 2 Institute of Medical Science, Faculty of Medicine, University of Toronto, Toronto, ON M5S 1A8, Canada; 3 Human Biology Department, University of Toronto, Toronto, ON M5S 3J6, Canada; sabah.kahnemuyipour@mail.utoronto.ca; 4 Department of Surgery, Faculty of Medicine, University of Toronto, Toronto, ON M5T 1P5, Canada

**Keywords:** spinal cord injury, microarray, cross-species comparison, transcriptional analysis

## Abstract

Despite the debilitating consequences following traumatic spinal cord injury (SCI), there is a lack of safe and effective therapeutics in the clinic. The species-specific responses to SCI present major challenges and opportunities for the clinical translation of biomolecular and pharmacological interventions. Recent transcriptional analyses in preclinical SCI studies have provided a snapshot of the local SCI-induced molecular responses in different animal models. However, the variation in the pathogenesis of traumatic SCI across species is yet to be explored. This study aims to identify and characterize the common and inconsistent SCI-induced differentially expressed genes across species to identify potential therapeutic targets of translational relevance. A comprehensive search of open-source transcriptome datasets identified four cross-compatible microarray experiments in rats, mice, and salamanders. We observed consistent expressional changes in extracellular matrix components across the species. Conversely, salamanders showed downregulation of intracellular MAPK signaling compared to rodents. Additionally, sequence conservation and interactome analyses highlighted the well-preserved sequences of *Fn1* and *Jun* with extensive protein-protein interaction networks. Lastly, in vivo immunohistochemical staining for fibronectin was used to validate the observed expressional pattern. These transcriptional changes in extracellular and MAPK pathways present potential therapeutic targets for traumatic SCI with promising translational relevance.

## 1. Introduction

Traumatic spinal cord injury (SCI) results in lifelong sensorimotor and autonomic disability, which is associated with severe physical, psychological, and socio-economic consequences for affected individuals and their families [1]. The incidence rate of traumatic SCI varies according to geographic location [2], but in the United States alone, it is estimated that there are approximately 17,900 new cases per year [3,4]. Traumatic SCI results from sudden mechanical damage to spinal neuronal, glial, and vascular cells due to an external force, and leads to a cascade of secondary molecular changes that further damage the spinal cord [5]. This secondary response is characterized mainly by early hypoxia, hemorrhage, and inflammation, which over-time results in cavitation and scar formation [6,7].

Traumatic SCI is a heterogeneous condition and demonstrates great cross-species variation [8,9]. Current treatments for SCI patients in the acute setting are limited to surgical decompression, augmentation of blood pressure to maintain cord perfusion, and acute neuroprotection with methylprednisolone sodium succinate (MPSS) [10]. As a systemic corticosteroid regimen, early MPSS administration can increase the risk of pneumonia and infection in SCI patients. Hence, MPSS is felt to have a relatively narrow margin of safety and is only efficacious if administered within eight hours post-injury [10]. Currently, there are several therapeutic candidates in clinical trials that hold promise to enhance the treatment options available to SCI patients [1]. However, SCI clinical trials often demonstrate low success rates both in safety and effectiveness [11]. Importantly, many molecular pathways involved in the secondary SCI response, such as inflammation, show great variation even across closely related species [12]. The elucidation of cross-species variation in SCI responses is crucial for the identification of highly conserved therapeutic targets [13]. In parallel, comparison to species that possess regenerative capabilities after SCI, such as salamanders and lampreys, allows for the characterization of novel regenerative signaling pathways after injury [14,15,16,17,18].

The recent proliferation of transcriptional data can provide valuable insights into species-specific SCI responses and the molecular pathways involved [15,19,20,21,22,23,24]. However, despite the great advances in transcriptome profiling in preclinical SCI models, a detailed understanding of how SCI responses compare and contrast across species remains elusive. The objective of this study is to identify and characterize both common and inconsistent SCI-induced differentially expressed genes (DEGs) across species to explore therapeutic targets with a higher chance of clinical translation. Given the importance of early intervention on the progression of secondary injury [5,25,26], this study will focus on the early subacute injury-time point (between 3/4-days post-SCI).

This study utilizes open-source transcriptome profiles from the Gene Expression Omnibus (GEO) database and uses g:Profiler to perform Gene Ontology (GO) and Kyoto Encyclopedia of Genes and Genomes (KEGG) pathway enrichment analyses [25,26,27]. This is the first study to compare SCI-induced transcriptional changes across the three species of rat, mouse, and salamander, as well as validating the results using in vivo immunohistochemical analysis. In addition, this is the first study to investigate the sequence conservation across common traumatic SCI animal models using open-source genomic databanks. The findings of this study will (i) further our understanding of early subacute secondary SCI pathophysiology across species, (ii) highlight novel therapeutic targets that have an increased chance of translating across species, and (iii) identify regenerative targets in the injured spinal cord.

## 2. Results

**Figure 1:**
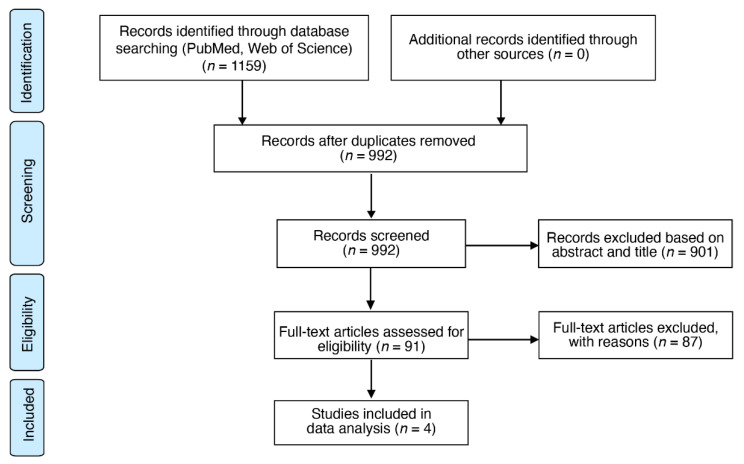
Search strategy flowchart. A PRISMA flowchart summarizing the search strategy used to identify articles with open-source SCI transcriptome databases in rats, mice, and salamanders [30]. A total of four cross-compatible microarray studies were isolated to be included in the final analysis.

This study isolated and compared four cross-compatible microarray analyses in rats, mice, and salamanders (Mexican axolotls) following traumatic SCI to explore novel therapeutic targets. The specified search strategy identified a total of 1159 articles. After duplicate removal and preliminary screening using inclusion/exclusion criteria, 91 articles remained for full-text assessment. Of the 91 articles, four cross-compatible microarray experiments in rats (GSE45006 [23]; GSE69334 [28]), mice (GSE34430 [29]), and salamanders (GSE71934 [15]) met all inclusion and exclusion criteria, which were subsequently included in the final analysis. Characteristics of each study were evaluated, and a quality assessment check was performed using the Collaborative Approach to Meta-Analysis and Review of Animal Data from Experimental Studies (CAMARADES) quality checklist (Table 1 and Table S1, respectively). A PRISMA flowchart outlining the search pipeline highlights the four cross-compatible microarray studies (Figure 1) [30].

### 2.1. Identification of Enriched GO Categories

**Figure 2:**
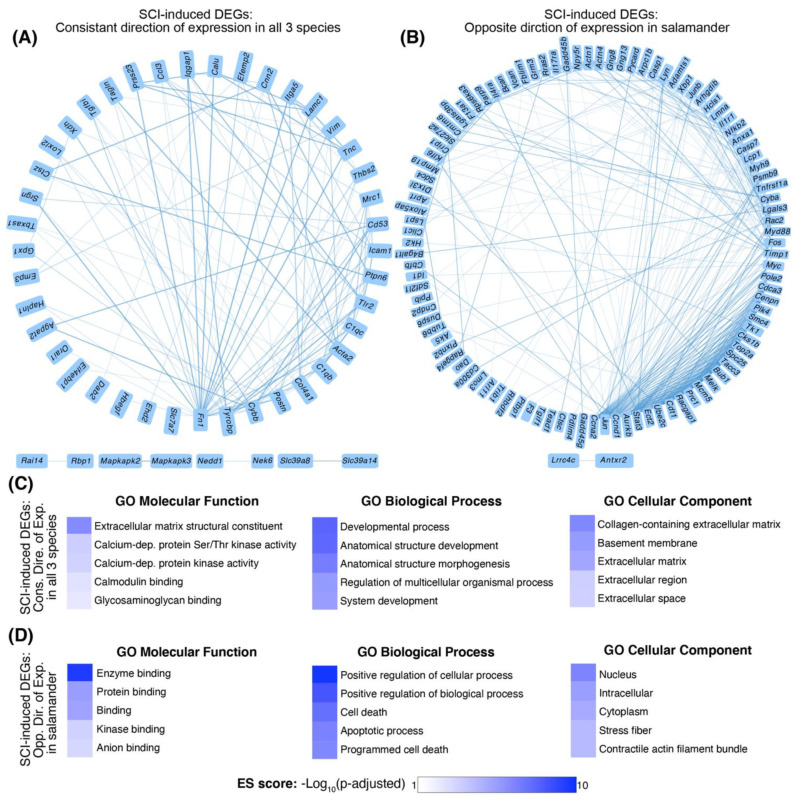
GO enrichment analysis of SCI-induced DEGs at 3/4 dpi. (**A**) The top five GO-Molecular Functions (MF), GO-Biological Processes (BP), and GO-Cellular Components (CC) enrichment scores for the 61 orthologs with a consistent direction of expression in all three species. The most enriched terms include those belonging to the extracellular matrix constituent (*p*-adj. = 1.10 × 10^−6^, ES score = 5.96) and development (“anatomical structure development”, *p*-adj. = 4.78 × 10^−8^, ES score = 7.32). (**B**) GO-MF, GO-BP, and GO-CC enrichment scores for the 119 orthologs with an opposite direction of expression in salamanders. The most enriched terms are related to intracellular components (“nucleus”, *p*-adj. = 6.44 × 10^−7^, ES score = 6.19) and positive regulation of apoptotic processes, such as “cell death” (*p*-adj. = 8.56 × 10^−8^, ES score = 7.06). Enrichment scores are calculated as the -log_10_ of the adjusted *p*-value. (**C**,**D**) STRING protein-protein network analysis in Cytoscape app. (**C**) Network analysis for SCI-induced DEGs with the consistent direction of expression in all four studies. (**D**) Network analysis for SCI-induced DEGs with opposite directions of expression in salamanders.

A total of 198 orthologs were significantly differentially expressed (*p*-adj. ≤ 0.05) in the injured animals compared to their uninjured controls in all four studies at 3/4-days post-injury (dpi) (Table S2). Of the 198, 61 orthologs had a consistent direction of expression across three species (either up- or down-regulated in all three species), and 119 had an opposite direction of expression in salamander (e.g., down-regulated in salamander but up-regulated in rats and mice or vice versa). Eighteen orthologs did not have either of the above expressional patterns and were excluded from the analysis. The majority of the orthologs with a consistent direction of expression were upregulated in all three species (Figure 2A, Table S2). Conversely, orthologs with an opposite direction of expression in the salamander were mainly downregulated in salamanders but upregulated in rats and mice (Figure 2B, Table S2).

We performed a full GO enrichment analysis of “Molecular Function (MF)”, “Biological Process (BP)”, and “Cellular Component (CC)” using the 61 and 119 orthologs separately (Figure S1). For the 61 orthologs with a consistent direction of expression across all three species, the most enriched GO-MF and GO-CC terms were related to the extracellular matrix (ECM), such as “extracellular matrix structural constituent” (*p*-adj. = 1.10 × 10^−6^, ES score = 5.96) or “collagen-containing ECM” (*p*-adj. = 7.94 × 10^−7^, ES score = 6.10) (Figure 2C). The most enriched terms within GO-BP were related to development, such as “anatomical structure development” (*p*-adj. = 4.78 × 10^−8^, ES score = 7.32) (Figure 2C).

We observed an opposite GO enrichment pattern for the 119 orthologs with a different direction of expression in salamanders. The top 5 GO-CC terms for these orthologs were all related to intracellular as opposed to extracellular functions (Figure 2D), including “nucleus” (*p*-adj. = 6.44 × 10^−7^, ES score = 6.19) and “cytoplasm” (*p*-adj. = 2.18 × 10^−5^, ES score = 4.66). Similarly, the top 5 GO-BP terms for these orthologs were all related to positive regulation of apoptotic processes as opposed to anatomical development, including “cell death” (*p*-adj. = 8.56 × 10^−8^, ES score = 7.06) (Figure 2D). The most enriched term in GO-MF was that of “enzyme binding” (*p*-adj. = 6.21 × 10^−10^, ES score = 9.21).

### 2.2. Identification of Enriched KEGG Pathways

**Figure 3:**
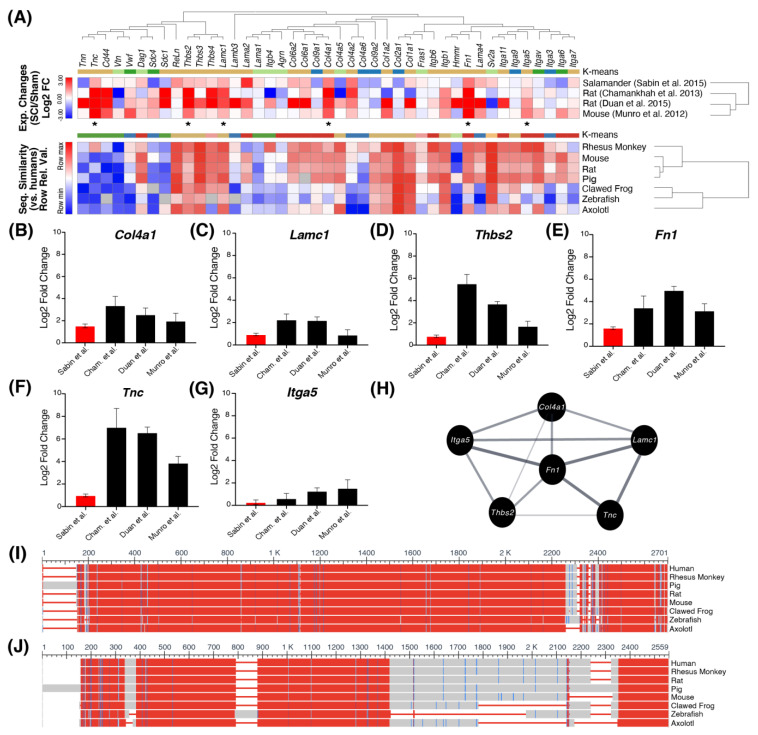
ECM-receptor interaction pathway (KEGG ID: mmu04512) contained the greatest number of orthologs with consistent direction of expression in all four studies (*p*-adj. = 0.003, ES score = 3.51). (**A**) Orthologs that were expressed in all four studies were included in the top heatmap, and *Mus musculus* was used as the reference organism. Asterisks (*) indicate genes that were significantly differentially expressed after SCI (*p*-adj. ≤ 0.05) in all four studies. Based on cosine similarity, the species were hierarchically clustered, and the genes were classified via K-Means into four groups. Additionally, the genes were ordered according to evolutionary distances as determined by the phylogenetic tree, which was constructed via the neighbor-joining method in MEGA X. The bottom heatmap compares the relative amino acid percent sequence identity of each gene across the species commonly used in SCI studies. K-means based on cosine similarity was used to classify the bottom heatmap into six groups. (**B**–**G**) The selected bar graphs depict the log2FC values for orthologs that were significantly differentially expressed after SCI in all four studies (*p*-adj. ≤ 0.05), including in salamanders (depicted in red). Data represented in Log2FC ± 95% confidence interval. (**H**) The protein-protein interaction for the six SCI-induced DEG with consistent direction of expression. (**I**,**J**) COBALT multiple sequence alignment for fibronectin 1 and tenascin c proteins, respectively. Conserved amino acid sequences are shown based on the relative entropy threshold of the residue. Red indicates high conservation and blue indicates lower conservation, whereas grey indicates amino acids in positions that are not conserved. Only regions with no gaps are colored.

Next, we performed a KEGG pathway enrichment analysis using the 61 and 119 orthologs. In concordance with our GO enrichment results, the top-most affected KEGG pathway for the 61 orthologs with a consistent direction of expression was “ECM-receptor interaction” (KEGG ID: mmu04512, *p*-adj. = 0.003, ES score = 3.51) (Figure 3A). The orthologs within this pathway that were significantly differentially expressed after SCI (*p*-adj. ≤ 0.05) in all four studies include *Col4a1* (collagen-4, alpha chain), *Lamc1* (laminin gamma subunit), *Thbs2* (thrombospondin-2), *Fn1* (fibronectin-1), *Tnc* (tenascin-C), and *Itga5* (integrin subunit alpha-B) (Figure 3B–G). All six of these orthologs were consistently upregulated across the four studies (ranging from 0.27 to 7.03 log2FC). Additionally, *Fn1* has an extensive protein-protein interactome and a well-preserved sequence across species (Figure 3H–J).

**Figure 4:**
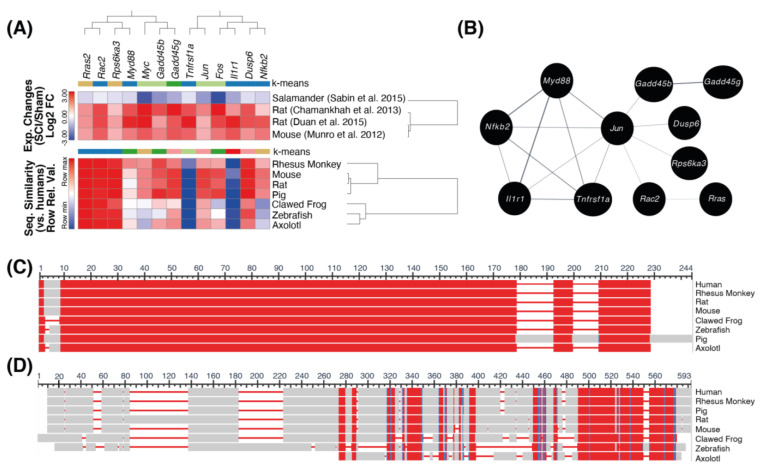
KEGG pathway enrichment analysis of orthologs with an opposite direction of expression in salamanders at 3 dpi. (**A**) Heatmap illustrating the MAPK signaling (KEGG ID: mmu04010) genes that contain the highest number of DEGs with opposing directionality in salamanders (*p*-adj. = 6.01 × 10^−5^, ES score = 4.22). Only orthologs that were expressed in all four studies were included in the heatmap. *Mus musculus* was used as the reference organism. Species were hierarchically clustered based on the cosine similarity metric of the average linkage method. Genes were divided into four groups based on the one minus cosine similarity method of K-Means clustering. Additionally, the genes were ordered according to evolutionary distances as determined by the phylogenetic tree, which was constructed via the neighbor-joining method in MEGA X. Only genes that were significantly differentially expressed after SCI (*p*-adj. ≤ 0.05) in all four studies are represented. The bottom heatmap compares the relative amino acid percent sequence identity of each gene across species commonly used in SCI studies. K-means based on cosine similarity was used to classify the bottom heatmap into six groups. (**B**) The protein-protein interaction for eleven of the SCI-induced DEGs with opposite directions of expression in salamanders. (**C**,**D**) COBALT amino acid sequence alignment of Rras2 and Tnfrsf1a, respectively. Conserved amino acid sequences are shown based on the relative entropy threshold of the residue. Red indicates high conservation and blue indicates lower conservation, whereas grey indicates amino acids in positions that are not conserved. Only regions with no gaps are colored.

For the 119 orthologs with a different direction of expression in salamanders, the top-most affected pathway was that of “mitogen-activated protein kinase (MAPK) signaling” (KEGG ID: mmu04010, *p*-adj. = 6.01 × 10^−5^, ES score = 4.22) (Figure 4A and Figure S2). The orthologs in this pathway that were significantly differentially expressed after SCI (*p*-adj. ≤ 0.05) in all four studies include *Rras2* (Ras-related protein, R-Ras2), *Rps6ka3* (ribosomal protein S6 kinase), *Myc* (Myc proto-oncogene), *Fos* (Fos proto-oncogene), *Tnfrsf1a* (TNF receptor superfamily member 1A), *Il1r1* (interleukin-1 receptor type 1), *Rac2* (Rac family small GTPase 2), *Myd88* (myeloid differentiation primary response protein), *Gadd45b* (growth arrest and DNA damage-inducible protein), *Gadd45g*, *Jun* (Jun proto-oncogene), *Dups6* (dual specificity phosphatase 6), and *Nfkb2* (nuclear factor kappa beta subunit 2) (Figure 4A). Across the four studies, these 13 orthologs were consistently downregulated in salamanders, and upregulated in rats and mice (with log2FC values ranging from −0.23 to 4.69). Within these orthologs, *Jun* shows the greatest protein-protein interaction network (Figure 4B).

### 2.3. Cross-Species Sequence Analysis

The percent amino acid sequence identity for the ECM and MAPK pathway orthologs was obtained from Protein BLAST with human genes as the reference. The results indicate that five genes in the ECM-related SCI-induced DEGs (*Col4a1*, *Lamc1*, *Thbs2*, *Fn1,* and *Itga5*) are highly conserved across mammals, ranging from 88–98% (Figure 3A). Out of these genes, *Thbs2* and *Lamc1* are the most conserved in salamanders (77.4% and 79.5%, respectively). *Fn1* was also highly conserved in salamanders with 75.4% identity similar to humans (Figure 3I). Although *Tnc* expression was upregulated in rats, mice, and salamanders, the *Tnc* gene is not well-conserved across species (Figure 3J). In contrast, for the MAPK pathway the amino acid sequences of *Rras2*, *Rps6ka3* and *Rac2* were the most conserved across species, ranging from 93–96% identity in salamanders (Figure 4A). *Gadd45b*, *Gadd45g*, *Jun,* and *Dusp6* were highly conserved across mammals (~95%) and 70–80% conserved in salamanders. *Myd88*, *Myc*, *Fos*, *Nfkb2*, *Tnfrsf1a,* and *Il1r1* were poorly conserved in clawed frogs, zebrafish, and axolotls, with *Tnfrsf1a* and *Il1r1* being the least conserved (33% and 37% in salamanders, respectively).

### 2.4. Immunohistological Validation of Fibronectin Expression

**Figure 5:**
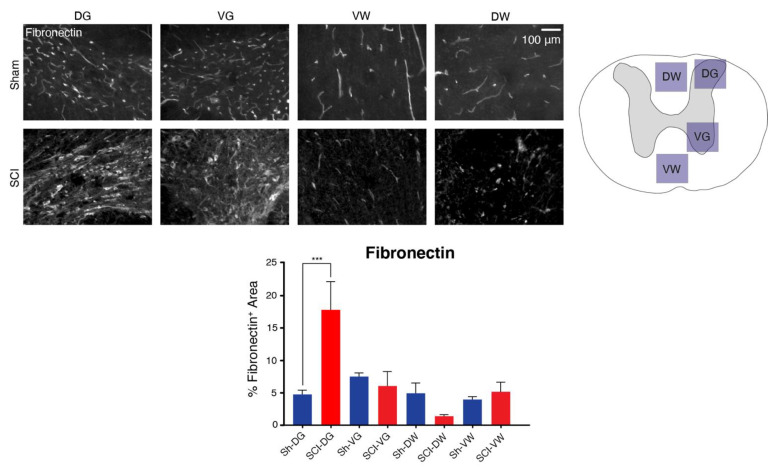
The presence of fibronectin (expressed by *Fn1*) in Wistar rat thoracic spinal cords at 3 dpi compared to time-matched laminectomized shams (*n* = 5 per cohort). The images were taken at the dorsal horn grey (DG), ventral horn grey (VG), dorsal horn white (DW), ventral horn white (VW) matter. Data represented as mean +/− SEM and analyzed using two-way ANOVA with Holm-Sidak multiple comparison test. *** indicates *p*-adj. < 0.001.

Fibronectin is a glycoprotein expressed by the *Fn1* gene and is significantly upregulated post-SCI in all four studies. Additionally, it has an extensive protein-protein interaction network and a well-preserved amino acid sequence across species (Figure 3H,I). In order to validate the consistent cross-species upregulation of fibronectin at the early subacute phase of secondary SCI, we stained the thoracic cords for both uninjured laminectomized shams and T6/7 clip-compression SCI in Wistar rats at 3-days post-operation (Figure 5). Four images were taken of the epicenter sections at the ventral grey matter (VG), dorsal grey matter (DG), ventral white matter (VW), and dorsal white matter (DW). The results demonstrate significant upregulation of fibronectin in the dorsal grey matter (−13.06 ± 2.733, *p*-adj: 0.0002; two-way ANOVA with Holm-Sidak multiple comparison), which further supports its upregulation after traumatic SCI.

## 3. Discussion

This is the first study to isolate and compare common and inconsistent SCI-induced DEGs in four cross-compatible microarray experiments in the salamander, rat, and mouse spinal cord, and to perform subsequent cross-species sequence conservation analysis. Although previous studies have highlighted differences across two species [19,31], the present study provides a comprehensive overview across multiple species at the early subacute phase. The results revealed that at 3/4 dpi, orthologs with a consistent direction of expression in all three species were mainly involved in ECM functioning and anatomical development. In contrast, orthologs with an opposite direction of expression in salamanders were predominantly involved in intracellular functions and apoptotic processes. As sequence conservation amongst common DEGs has significance for developing novel therapeutics, this study demonstrates that most ECM genes were highly conserved across common SCI animal models, whereas sequences in the MAPK pathway showed greater variability.

During the subacute phase of secondary SCI pathogenesis (2 to 14 dpi), endogenous repair of the blood spinal cord barrier (BSCB) takes place, which restores blood supply and augments further hypoxia, hemorrhage, and ischemic injury [6]. However, calcium dysregulation and other forms of ionic disturbance result in the overproduction of glutamate (known as glutamate excitotoxicity [32]), which leads to activation of a series of downstream protein kinases [33]. Additionally, the clearance of cellular debris by phagocytic inflammatory cells produces by-products such as free radicals that can cause lipid peroxidation, DNA oxidative damage, and protein oxidation [32]. Furthermore, the proliferation of perivascular fibroblast-derived stromal cells, meningeal fibroblasts, and pericytes leads to scar formation and modifications within the spinal ECM [7]. Treatment strategies targeting these early processes can have a lasting effect on functional recovery and chronic preservation of neural tissue [5,34], hence the present study aimed to further investigate therapeutic targets during the early subacute phase of injury.

The spinal ECM is crucial for maintaining structural and organizational integrity of the neural tissue, which consists of a hyaluronan backbone with tenascins and sulfated proteoglycan attachments that are either diffused in the interstitial space or densely accumulated around the neuronal cell bodies [35]. Following traumatic SCI, the released ECM molecules, such as hyaluronan fragments, tenascins, and sulfated proteoglycans, trigger inflammation and result in the release of degradative enzymes, such as matrix metalloproteinases (MMPs) [36]. Several studies have previously observed the differential expression of ECM constituents following SCI [22,37,38]. *Col4a1*, *Lamc1*, *Thbs2*, *Fn1*, *Itga5*, and *Tnc* code for classic components of the spinal ECM that interact with each other [36]. Our observation that these ECM entities are upregulated after SCI despite a difference in species or SCI model suggests that these structural changes and matrix remodeling patterns are highly preserved across species. However, it is unclear how each ECM molecule differentially affects the injury response in regenerative versus non-regenerative species. Nonetheless, our observations imply that ECM-focused drugs should be a top priority when trying to achieve interspecies translation. Treatments targeting the ECM components and perineuronal net, such as chondroitinase ABC therapy [37,38], have shown promising improvements in functional recovery after SCI [39,40,41] by degrading growth-inhibiting ECM entities like chondroitin sulfate proteoglycans (CSPGs) [37].

Fibronectin is a molecule shown to have both inhibitory and regenerative effects. Fibronectin is highly conserved and has important roles in tissue repair due to its adhesive properties and involvement in cellular differentiation/migration. On its own, plasma fibronectin has been shown to have neuroprotective effects [39]. However, it is also a commonly used marker for the fibrotic scar, which is inhibitory to axon regeneration. The fibrotic scar forms in the lesion core and consists of fibroblasts and ECM deposits. Our interactome analysis as well as previous studies on wound healing processes suggest that fibronectin is involved in regulating many ECM molecules, such as collagens, laminins, CSPGs, and tenascin-C [42]. In salamanders, fibrosis is temporary, and immune cells aid in matrix remodeling, leading to tissue restoration [31]. Therefore, it would also be worth investigating *Fn1* in the context of salamanders and mammals to see if there are specific cellular conditions that drive it towards a regenerative state rather than an inhibitory one.

Tenascins are a family of extracellular glycoproteins that interact closely with fibronectin [36]. In the healthy mammalian central nervous system (CNS), *Tnc* is only expressed in select regions [40]. However, after SCI, its expression peaks in astrocytes surrounding the lesion epicenter as well as within the epicenter itself [41]. Studies in mammals have shown that *Tnc* can have both inflammatory and regenerative functions [43,44,45]. Tenascin-C can recruit inflammatory components such as TLR-4 [43]. Schreiber et al. (2013) demonstrated that *Tnc* deficient mice show improved axonal plasticity after SCI [44]. However, work by Chen et al. (2010) highlights a beneficial role of *Tnc*, with mice lacking *Tnc* exhibiting worsened locomotor recovery compared to their wild-type counterparts, whilst *Tnc* restoration was able to rescue locomotor abilities [45]. In salamanders, *Tnc* has been shown to promote cell proliferation and migration during limb regeneration [46]. Given that *Tnc* has both inflammatory and regenerative functions, it is possible that the cellular conditions in salamanders drive *Tnc* towards its regenerative state (e.g., promoting axonal sprouting) as opposed to its inflammatory one. Further research involving the deletion of *Tnc* in regenerative species is needed to fully elucidate this hypothesis and to validate its mechanism of action after SCI.

For orthologs with a different direction of expression in salamanders, we observed an upregulation of MAPK signaling in rodents paired with a downregulation in salamanders. MAPK signaling is needed for the synthesis of cytokines in the innate immune system as well as activation of the adaptive immune system [47]. As Tica and Didangelos (2018) have noted in their analysis, downregulation of this pathway in salamanders may reflect a non-cytotoxic and weakened adaptive immune response [31,48]. In salamanders, scarless wound healing requires an intricate balance between pro- and anti-inflammatory signals [49]. Having a weakened adaptive immune response may assist in achieving this required balance. In contrast, the mammalian immune system is quite advanced [48] and can over-induce pro-inflammatory responses after injury. Excessive pro-inflammatory signaling destroys the delicate balance required for tissue regeneration [49].

In addition to MAPK signaling, orthologs with a different direction of expression in salamanders are also involved in other typical inflammatory pathways such as TNF signaling (*Tnfrsf1a*, *Jun*) [22,23], cytokine release (*Il1r1*) [23], toll-like receptor signaling (*Myd88*, *Il1r1*) [50] and NF-kappa B signaling (*Nfkb2*) [23]. The downregulation of inflammation in salamanders is likely due to their weakened adaptive immune response, which in part contributes to their regenerative abilities [48]. Other orthologs including *Myc*, *Rac2*, *Gadd45b*, and *Gadd45g* are involved in regulating apoptotic processes [22,24,51,52,53,54]. *Gadd45g* may be worth exploring as a novel therapeutic target. *Gadd45g* can regulate apoptotic and growth arrest processes [54], but its role after SCI has not been fully elucidated. However, our observation that it is downregulated in salamanders and upregulated in rats and mice between 3 and 4 dpi suggests that its expression may obstruct regeneration. Interestingly, low expression of *Gadd45g* has been associated with various cancers, indicating that it normally suppresses cellular growth and proliferation [55]. Therefore, suppressing the expression of *Gadd45g* in mammals after SCI may help to promote regeneration. Similar to *Tnc* and *Fn1*, further research is needed to confirm this hypothesis. Aside from the inflammatory genes, the function of two orthologs (*Rras2* and *Dusp6*) following SCI is poorly understood. *Rras2* and *Dusp6* are upregulated following sciatic nerve injury [56] and SCI [51] respectively, but their roles in each are not entirely clear.

Another point of interest is the upregulation of *Fos* and *Jun* in mammals paired with a downregulation in salamanders. In accordance with past research [24,51], we observed that both *Fos* and *Jun* are upregulated in mammals between 3 and 4 dpi. In mammals, c-Fos and c-Jun proteins combine to form the AP-1 transcription complex [57]. In the context of mammalian SCI, the AP-1 complex can contribute to the formation of glial scars [24], thereby explaining why the two orthologs are concurrently upregulated after injury. However, the AP-1 complex in salamanders is composed of c-Fos and JunB, in contrast to the mammalian variant of c-Fos and c-Jun [58]. In addition, the AP-1 complex in the ependymoglial cells of salamanders promotes spinal cord regeneration as opposed to glial scar formation at 1 dpi [15]. *Fos* is therefore typically upregulated in salamanders following SCI [15]. We did not, however, observe an upregulation of *Fos* at day 3. This may be because *Fos* is mainly an immediate-early response gene (e.g., upregulated at 30 min after injury [24]). Therefore, it is possible that by day 3 the expression levels of c-Fos are not as robust. We did, however, observe a downregulation of *Jun* (c-Jun) in salamanders at 3 dpi. In fact, Sabin et al. (2019) demonstrated that after SCI, salamanders express a miRNA—miR-200a—in glial cells to block the expression of c-Jun specifically [58]. If c-Jun is allowed to be expressed via inhibition of miR-200a, axonal regrowth and regeneration are greatly inhibited. Thus, mammalian and salamander AP-1 appear to have opposing functions in terms of regeneration. Future research may consider converting the mammalian AP-1 complex to contain the salamander c-Fos and JunB variants to see if regeneration after SCI can be improved.

While many studies have previously compared salamanders to rodents in various conditions [22,59,60,61,62], there are advantages and disadvantages that need to be considered. First, these species are evolutionarily distant and due to the variation in morphology, the injury model would differ between species. However, while the four studies included in our comparison differ in injury model and were performed by four different labs, the consistencies in expressional changes between these studies further signifies the therapeutic potential of the identified candidates. In contrast, comparison to salamanders will suggest potential regenerative targets. Second, while transcriptional analyses are powerful and informative, they only provide a snapshot of ongoing cellular processes. Hence, the immunohistological analysis is an important validation of the transcriptional analysis. Third, in comparison to RNA sequencing, microarray studies have a low sensitivity for gene expression (e.g., it cannot detect transcripts that are expressed at very low or very high levels) [63]. Thus, the studies included in our analysis may not reflect the full transcriptome after SCI. Lastly, this type of analysis cannot provide any single-cell information and the cell types involved in SCI-induced transcriptional changes. Future research with single-cell RNA sequencing can provide important insights into the cell-specific injury response.

## 4. Materials and Methods

### 4.1. Systematic Review

Two electronic databases (PubMed, Web of Science) were thoroughly searched from inception to 6 May 2020. Medical Subject Headings (MeSH) and keywords were used on PubMed and Web of Science, respectively. The following search strategy was employed: (spinal cord injury AND (gene expression profil* OR transcriptome OR sequencing OR RNA sequencing OR microarray) AND lamprey/salamander/rat/mouse/porcine/non-human primates). Articles were limited to those published in English. In order to identify PubMed articles that have not yet been categorized with a MeSH term, a free search using the following terms: “spinal cord injury”, “RNA sequencing”, “lamprey”, “salamander”, “porcine”, “rat”, “mouse”, and “non-human primates” was also conducted. All search results were exported to Mendeley for duplicate removal.

The selected articles had to contain openly accessible transcriptome data in the NCBI GEO database with an indication of the sample size to be included in the final analysis. The data extraction was focused on the early subacute phase (3- to 4-days post-SCI) in the thoracic spinal cord. Selected articles also had to contain either an uninjured sham or a naive control. Studies reporting non-traumatic SCI or in vitro models were excluded. Furthermore, studies were excluded if the microarray normalization method used deviated from the commonly used Robust Multi-chip Average (RMA) or Guanine Cytosine Multi-Chip Average (gcRMA) methods. The Collaborative Approach to Meta-Analysis and Review of Animal Data from Experimental Studies (CAMARADES) quality checklist was used to perform a quality assessment on the selected articles [64].

### 4.2. Microarray Data

Four pre-normalized gene expression profiles (GSE34430 [29], GSE45006 [23], GSE69334 [28]; and GSE71934 [15]) were downloaded from the NCBI GEO database [25] using the GEOquery package (v.2.56.0) [65] of R/Bioconductor [66]. Briefly, Chamankhah et al. (2013) (GSE45006) induced SCI at T7 in Wistar rats using a clip-compression model. RNA was collected for microarray at 1, 3, 7, 14, and 56 dpi. Four injured animals per time-point were used in addition to four sham animals. Sabin et al. (2015) (GSE71934) performed an SCI transection in axolotls and collected RNA at 1, 3, and 7 dpi. A total of three replicates existed per time point, with 10 axolotls pooled into each replicate. Three control replicates were also used. Duan et al. (2015) (GSE69334) induced SCI at T7-T8 in Wistar rats using a transection model and collected RNA at 1, 3, 10, 20, 30, 60, and 90 dpi. Three injured replicates existed per time-point, with four rats pooled into each replicate. Three uninjured replicates were also used as controls. Munro et al. (2012) (GSE34430) performed a left-side hemisection at T13-L1 in C57/Bl6 mice and collected RNA at 4 dpi. A total of three injured and three sham animals were used. The present study only used injured samples from 3 and 4 dpi as well as their respective sham/naïve controls. The RNA samples included in the present analysis were all collected at the lesion epicenter. Those not from the epicenter were excluded from the analysis. Table 1 summarizes the characteristics of each study.

### 4.3. Differential Gene Expression Analysis

The Limma package (v3.44.1) [67] within R/Bioconductor [66] was used to isolate differentially expressed genes. For each study, pairwise comparisons were made between the SCI and uninjured/sham groups. For probesets that mapped onto the same gene, the probeset with the highest average expression was taken as the representative. P-values were adjusted for multiple comparisons using the Benjamini-Hochberg false discovery rate procedure. A table containing gene names, their log2 fold-change values (log2FC), adjusted *p*-values, and confidence intervals was exported for each dataset.

Gene annotations for Chamankhah et al. (2013) [23] and Duan et al. (2015) [28] were added using the annotate [68] package (v1.66.0) of Bioconductor. Annotation files for Munro et al. (2012) [29] were obtained from the GEO website under platform number GPL6193 (https://www.ncbi.nlm.nih.gov/geo/query/acc.cgi?acc=GPL6193, accessed on 31 May 2020). Annotation files for Sabin et al. (2015) [15] can be found at Sal-Site (https://ambystoma.uky.edu/quick-links/microarray-database, accessed on 30 May 2020).

### 4.4. Ortholog Mapping

In order to make the genes comparable across three different species, the genes in each species were mapped onto mouse orthologs using g:Orth of g:Profiler [27]. Axolotl gene annotations were already mapped onto human orthologs [69] and were thus further converted into mouse orthologs for comparison with rodent species. If one gene mapped onto several orthologs, then the top ortholog was kept. Only orthologs that were present in all three species were kept for the final analysis.

### 4.5. Sequence Conservation Analysis

Percent amino acid sequence conservation for the SCI-induced DEGs in both the ECM and MAPK pathways was used to assess the translatability of these therapeutic targets across species. Amino acid sequences (FASTA format) for the genes of interest in humans and the commonly used SCI animal models, including mice (*Mus musculus*), rats, clawed frogs (*Xenopus tropicalis*), pigs (*Sus scrofa*), and zebrafish (*Danio rerio*), were extracted from the NCBI Reference Sequence database [70]. Salamander sequences were extracted from the UCSC Genome Browser database [71,72]. In the case where a gene has multiple sequences, the longest isoforms were chosen [73]. If a gene existed in two locations in the salamander genome, the one most similar to *Xenopus tropicalis* was chosen. The percent identity for each gene was established by using Protein BLAST to compare the orthologs to humans, depicted on heatmaps created through Morpheus (Broad Institute, Cambridge, MA, USA, https://software.broadinstitute.org/Morpheus, accessed on 22 July 2020). Species were hierarchically clustered based on cosine similarity. Additionally, multiple alignments were retrieved from the Constraint-based Multiple Alignment Tool (COBALT) with default parameters to show conservation [74]. To construct phylogenetic trees, multiple sequence alignment was also done in Molecular Evolutionary Genetics Analysis X (MEGA X) using the MUSCLE algorithm, which was set up with default parameters [75,76]. The phylogenetic tree was constructed in MEGA X using the neighbor-joining method. The evolutionary distances were computed using the Poisson correction method and are in the units of the number of amino acid substitutions per site. All ambiguous positions were removed for each sequence pair (pairwise deletion option).

### 4.6. Ontological and Pathway Enrichment Analyses

Orthologous genes with adjusted *p*-values ≤ 0.05 in all four studies were considered to be differentially expressed. Among the differentially expressed orthologs, those that had a consistent direction of expression across the three species (e.g., up-regulated in all three) and those that had an opposite direction of expression in salamander (e.g., down-regulated in salamander but up-regulated in rats and mice or vice versa) were entered separately into g:Profiler for Gene Ontology and KEGG pathway enrichment analysis. *Mus musculus* was used as the reference organism. A cut-off of *p*-adjusted ≤ 0.05 was used to filter for significantly affected GO and KEGG pathways.

### 4.7. Interactome Analysis

All significantly expressed SCI-DEGs with a consistent direction of expression across species or inconsistent direction of expression in salamanders were used for interactome analysis. The protein-protein network interaction was completed in Cytoscape 3.8.2. using the STRING dataset. The interactome pathways were shown using “degree sorted circle layout”. *Mus musculus* was used as a reference. The confidence score cut-off was set to 0.4. Singletons were removed from the analysis.

#### 4.7.1. GO Enrichment Analysis

The GO categories of “Molecular Function (MF)”, “Biological Process (BP)”, and “Cellular Components (CC)” were analyzed for significantly enriched terms. GraphPad Prism Version 8.4.3 (GraphPad Software Inc., La Jolla, CA, USA, www.graphpad.com, accessed on 15 July 2020) was used to generate heatmaps of the top five most enriched terms within each GO category. Enrichment scores as well as the number and percentage of query genes that fell into each GO category were plotted.

#### 4.7.2. KEGG Analysis

The top-most affected non-disease KEGG pathways were isolated for orthologs with a consistent direction of expression in all three species, as well as those with a different direction of expression in the salamander. Morpheus was used to generate heatmaps of the genes in each pathway [77]. Only orthologs that had an expression value in all four studies were included. If the ortholog was missing in any of the four studies, it was excluded from the heatmap. Finally, orthologs were marked with an asterisk if they were significantly differentially expressed (*p*-adj. ≤ 0.05) in the injured animals compared to their uninjured/sham controls in all four studies. Species were hierarchically clustered based on one minus cosine similarity metric of the average linkage method. Genes were sorted into four clusters using the one minus cosine similarity method of K-Means clustering.

### 4.8. Animal Care and Surgery

All animal work was performed on 12-week-old inbred Wistar rats weighing between 230g to 280g (obtained from Charles River Laboratories, Wilmington, MA, www.criver.com). The daily care was in compliance with the Canadian Council on Animal Care and the committee at the University Health Network (UHN). The Wistar rats underwent either a laminectomy at T6–7 (sham) or a 35-gram clip compression injury for 1 min [78]. Five percent inhalant isoflurane in O2 carrying gas was used to anesthetize the rats, and 2% isoflurane was used to maintain anesthesia throughout the surgeries. Rats were subcutaneously injected with 0.05mg/kg of buprenorphine and 5mL of saline immediately post-surgery. At 3 dpi, the animals (*n* = 5 for SCI, *n* = 3 for sham) were sacrificed and fixed with 4% *w*/*v* paraformaldehyde (PFA) in phosphate-buffered saline (PBS). The spinal cord was exposed and a 1 cm segment of the spinal cord, centered on the injury epicenter or 1 cm from the T6 lamina (sham), was removed and post-fixed in 10% *w*/*v* sucrose in 4% PFA in PBS for 4 h. The cord was then transferred to 20% sucrose, embedded in the Epredia™ M1 Embedding Matrix (1310, www.fishersci.com), and snap-frozen on dry ice.

### 4.9. Immunohistological Analysis

The extracted spinal cord was mounted for cryosectioning and cut cross-sectionally at a thickness of 30 microns. Tissues were then stained with the MilliporeSigma anti-fibronectin primary antibody (1:500, F3648, www.sigmaaldrich.com) followed by a Abcam goat anti-rabbit Alexa-488 fluorescent secondary (1:1000, ab150077, www.abcam.com). The coverslips were mounted in Mowiol and allowed to be set overnight. The slides were imaged on a Zeiss epifluorescent microscope at a magnification of 16x. Image analysis was performed in Fiji thresholding the fibronectin signal, binarizing the image, and measuring the area fraction of fibronectin+ staining [79].

## 5. Conclusions

This study compares and contrasts the SCI-induced transcriptional changes in three species between 3 and 4 dpi. Findings suggest an upregulation of ECM entities across all three species. The results also suggest the downregulation of internal signaling pathways in salamanders. These findings have important implications for developing SCI therapies with a higher chance of interspecies translation and identifying potential regenerative treatments.

## Figures and Tables

**Table 1:** Summary of the four cross-compatible microarray studies.

Author	Species	Injury Model	Sample Size	Time-Point (dpi)	Normalization Format
Chamankhah et al. (2013) [23]	Wistar rat	T7 Clip-Compression	4 injured rats/time point 4 sham rats	1, 3, 7, 14, and 56 days	gcRMA
Sabin et al. (2015) [15]	Axolotl (salamander)	Transection (Caudal to cloaca)	3 injured replicates/time point 3 uninjured replicates (10 axolotls were pooled into each replicate)	1, 3, and 7 days	RMA
Duan et al. (2015) [28]	Wistar rat	T7/8 Transection	3 injured replicates/time point 3 uninjured replicates (4 rats were pooled into each replicate)	1, 3, 10, 20, 30, 60, and 90 days	RMA
Munro et al. (2012) [29]	C57/Bl6 Mouse	T13/L1 Left hemisection	3 injured mice 3 sham mice	4 days	RMA

## Data Availability

The data presented in this study are contained within the article and supplementary materials.

## Supplementary Materials

The following are available online at https://www.mdpi.com/article/10.3390/ijms222312934/s1.
