# Supplementary material for: The MAPK Signaling Pathway Presents Novel Molecular Targets for Therapeutic Intervention after Traumatic Spinal Cord Injury: A Comparative Cross-Species Transcriptional Analysis
Source: Int J Mol Sci. 2021 Nov 29;22(23):12934. doi: 10.3390/ijms222312934 (PMC8657729; doi:10.3390/ijms222312934)
Supplement: Supplementary file 1 [file ijms-22-12934-s001.zip › ijms-1392741 proof done-supplementary.pdf]

International Journal of
Molecular Sciences

MDPI

Supplementary Material

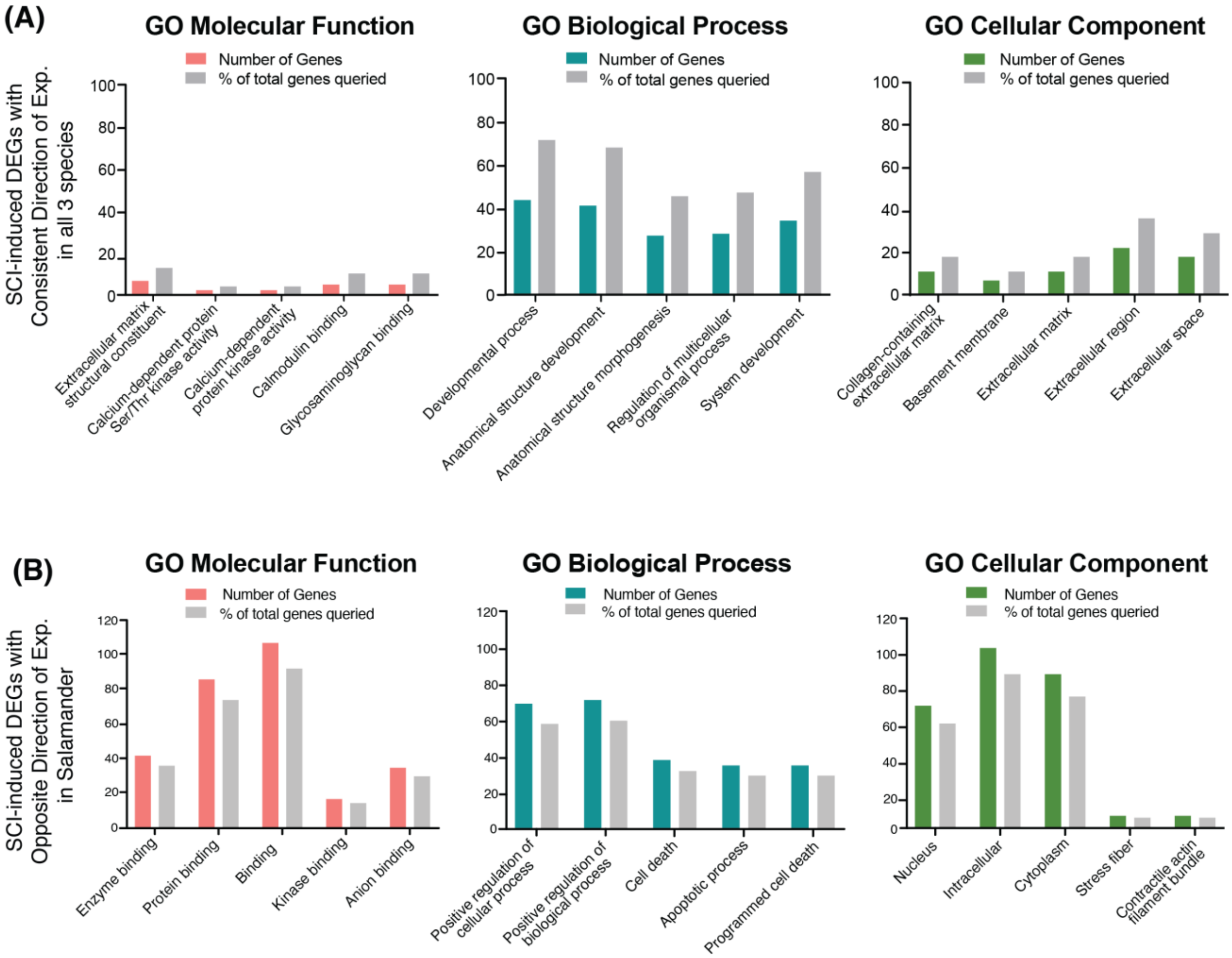

Figure S1. Distribution of SCI-induced DEGs in each GO category. (A) Bar graphs depict the number and percentage of query genes that fell into each GO category for orthologs with a consistent direction of expression in all 3 species. GO “Biological Process” had the highest number of genes overall. (B) The number and percentage of query genes in each GO category for orthologs with an opposite direction of expression in salamanders. All 3 categories had a relatively similar number of genes.

International Journal of
Molecular Sciences
MDPI

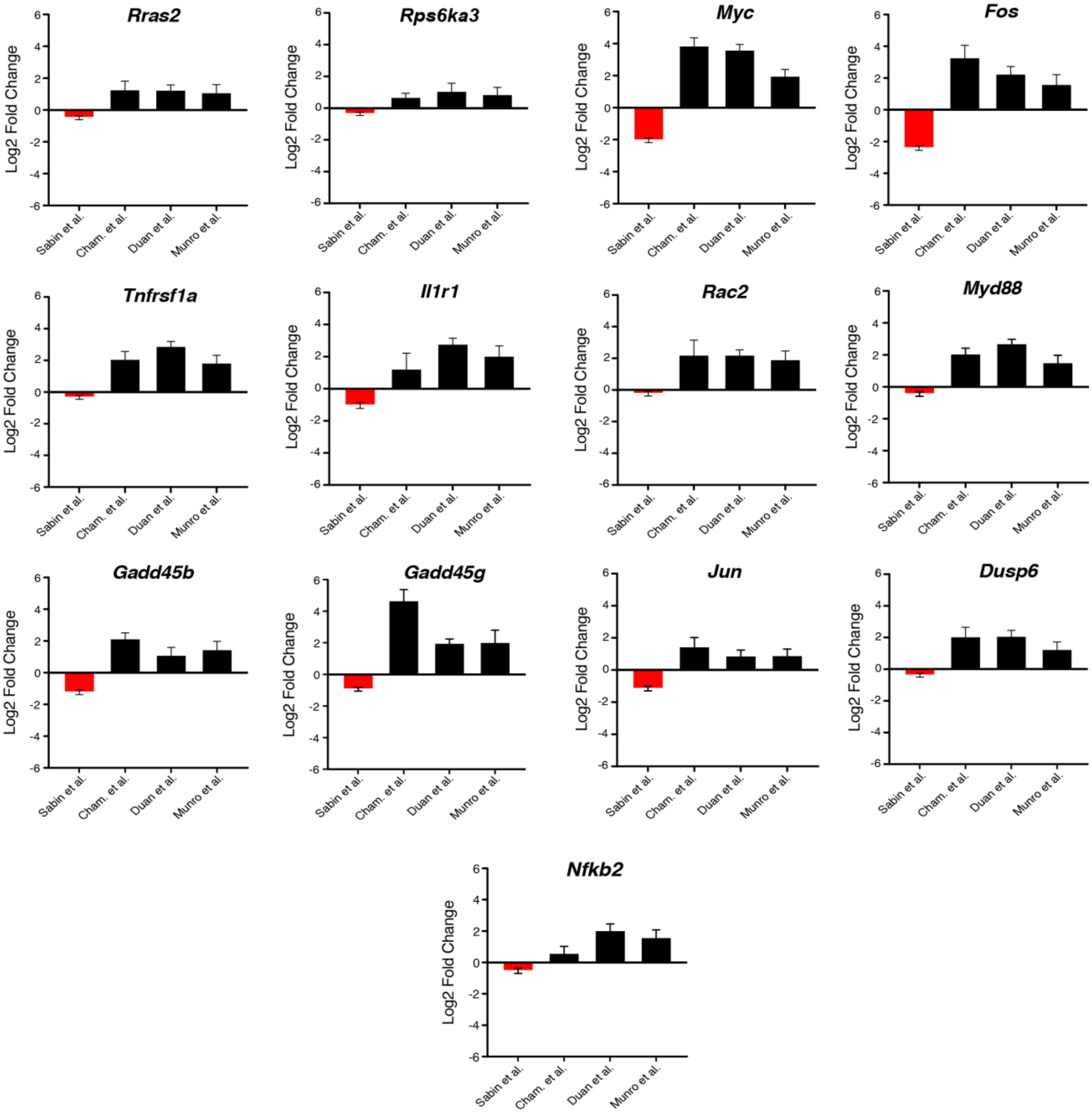

Figure S2. Log2FC Bar graphs for the MAPK pathway orthologs. These were significantly differentially expressed after SCI (p-adj. ≤ 0.05) in all four studies. The 13 orthologs (*Rras2*, *Rps6ka3*, *Myc*, *Fos*, *Tnfrsf1a*, *Il1r1*, *Rac2*, *Myd88*, *Gadd45b*, *Gadd45g*, *Jun*, *Dusp6*, *Nfkb2*) were downregulated in salamanders (red) and upregulated in rodents (black). Error bars indicate the 95% confidence interval.

International Journal of Molecular Sciences

MDPI

Table S1. Quality control checklist for studies included in the cross-species comparison.

Article	Peer Reviewed	Statement of Temperature Control	Randomization of Treatment/Control	Allocation concealment	Blinded assessment of outcome	Avoidance of anesthetics with marked intrinsic properties	Use of animals with hypertension or diabetes	Sample size calculation	Statement of compliance w/ regulatory requirements	Statement regarding possible conflict of interest
Chamankhah et al. (2012)	Y	N	*N/A	N/A	N/A	Halothane and a 1:1 mixture of O2/N2O	N	N	Y	Y
Sabin et al. (2015)	Y	Y (for immunohistochemistry)	N/A	N/A	N/A	0.01% p-amino benzocaine	N	N	Y	N
Duan et al. (2015)	Y	Y (for immunohistochemistry)	Y (for behavioural work)	N/A	Y (for behavioural work)	Intraperitoneal (IP) injections of 6% chloral hydrate	N	N	Y	Y
Munro et al. (2012)	Y	Y (for immunohistochemistry)	N/A	N/A	Y (for histology)	Intraperitoneal (IP) injection of ketamine and xylazine	N	N	Y	Y

*Note: "N/A" indicates that the measure was not explicitly commented upon within the article.

Table S2. List of orthologs with common or inconsistent expression patterns across the 3 species.

**Orthologs with Consistent Direction of Exp. in all 3 Species** (61 total)		**Orthologs with an Opposite Direction of Exp. in Salamander** (119 total)	
Upregulated in all 3 species	Downregulated in all 3 species	Downregulated in salamander, upregulated in rat & mice	Upregulated in salamander, downregulated in rat & mice
Spry1	Esrrg	Cdt1	Grm3
Postn	Hapln1	Aprt	Lrrc4c
Xdh		Tubb6	Lmo3
Hbegf		Xbp1	Dao
Thbs2		Trib1	Npy5r
Cnn2		Myc	Cbln1
Mvp		Adamts1	Olfm3
Tgfbi		Alox5ap	Rapgef4
Orai1		Gadd45g	Ak5
Atp6v0e		Emp1	Bcan
Surf4		Clic1	Gng8
Emp3		Myd88	Slc27a2
Lamc1		Gadd45b	Gng13
Tnc		Rbm47	
Hspb8		Il17ra	
Col4a1		Lsp1	
Calu		Tgif1	
Eif4ebp1		Midn	
Vamp8		Il4ra	
Mrc1		Racgap1	

International Journal of
Molecular Sciences

MDPI

Loxl2
Srgn
Prss23
Fn1
Rai14
Iqgap1
Rbp1
Gpx1
Tbxas1
Slc39a14
Mapkapk3
Ehd2
Nek6
Efemp2
C1qb
Tlr2
Acta2
Ctsz
Man1a
Icam1
C1qc
Dab2
Vim
Ccng1
Nln
Slc7a7
Steap4
Agpat2
Ptpn6
Gba
Slc39a8
Ccl3
Tagln
Cybb
Nedd1
Cd53
Mapkapk2
Itga5
Tyrobp

Lmna
Crip1
Klf6
Tnfrsf1a
Fos
Actn1
Stat3
Alg8
Cttnbp2nl
Pycard
Sdf2l1
Ccna2
Cks1b
Mmp19
Top2a
F13a1
Tacc3
Stk17b
Anxa1
Mcm5
Ctsc
Dusp6
Ptbp1
Timp1
Junb
Pdlim4
F3
Bub1
Casp1
Hcls1
Ftsj3
Ccnd1
Cenpn
Rps6ka3
Lcp1
Myh9
Cndp2
Antxr2
Jun
Arhgdib
Cmtm6
Prc1
Spc25
Id1
Vcan
Tk1
Rras2
Rac2
Ppib
Pmepa1
Lyn
Melk
B4galt1

International Journal of Molecular Sciences

	Cbfb
	Lgals3
	Tead1
	Elk3
	Npl
	Sgpl1
	Psmb9
	Tmpo
	Arpc1b
	Cyba
	Lgals3bp
	Smc4
	Rhbdf2
	Casp7
	Actn4
	Ube2c
	Dtx3l
	Cd300a
	Arl11
	Plxnb2
	Ect2
	Aurkb
	Cdca3
	Sdc4
	Fblim1
	Rgs19
	Nfkb2
	Hk2
	Parp9
	Rrbp1
	Pole2
	Plk4
	Il1r1
